# Lipo-Based Vaccines as an Approach to Target Dendritic Cells for Induction of T- and iNKT Cell Responses

**DOI:** 10.3389/fimmu.2020.00990

**Published:** 2020-05-27

**Authors:** Dorian A. Stolk, Aram de Haas, Jana Vree, Sanne Duinkerken, Joyce Lübbers, Rieneke van de Ven, Martino Ambrosini, Hakan Kalay, Sven Bruijns, Hans J. van der Vliet, Tanja D. de Gruijl, Yvette van Kooyk

**Affiliations:** ^1^Department of Molecular Cell Biology and Immunology, Amsterdam UMC, Cancer Center Amsterdam, Amsterdam Infection and Immunity Institute, Vrije Universiteit Amsterdam, Amsterdam, Netherlands; ^2^Department of Medical Oncology, Amsterdam UMC, Cancer Center Amsterdam, Amsterdam Infection and Immunity Institute, Vrije Universiteit Amsterdam, Amsterdam, Netherlands; ^3^Department of Otolaryngology/Head and Neck Surgery, Amsterdam UMC, Cancer Center Amsterdam, Amsterdam Infection and Immunity Institute, Vrije Universiteit Amsterdam, Amsterdam, Netherlands; ^4^LAVA Therapeutics, Utrecht, Netherlands

**Keywords:** dendritic cell, DC-SIGN, langerin, liposome, targeting, iNKT, cancer vaccine, T-cell priming

## Abstract

In this study we developed a liposome-based vaccine containing palmitoylated synthetic long peptides (SLP) and alpha galactosylceramide (αGC) to specifically target dendritic cells (DC) for activation of both innate (invariant natural killer T-cells [iNKT]) and adaptive (CD8^+^ T-cells) players of the immune system. Combination of model tumor specific antigens (gp100/MART-1) formulated as a SLP and αGC in one liposome results in strong activation of CD8^+^ and iNKT, as measured by IFNγ secretion. Moreover, addition of lipo-Lewis Y (Le^Y^) to the liposomes for C-type lectin targeting increased not only uptake by monocyte-derived dendritic cells (moDC), dermal dendritic cells and Langerhans cells but also enhanced gp100-specific CD8^+^ T- and iNKT cell activation by human skin-emigrated antigen presenting cells in an *ex vivo* explant model. Loading of moDC with liposomes containing Le^Y^ also showed priming of MART-1_26−35L_ specific CD8^+^ T-cells. In conclusion, chemically linking a lipid tail to a glycan-based targeting moiety and SLP combined with αGC in one liposome allows for easy generation of vaccine formulations that target multiple skin DC subsets and induce tumor antigen specific CD8^+^ T- and iNKT cells. These liposomes present a new vaccination strategy against tumors.

## Introduction

During the past decade substantial progress has been made in the field of cancer immunotherapy, especially with the rise of immune checkpoint inhibitor (ICI) therapy (1–3). Unfortunately, as for many other therapies, the amount of patients that respond to ICI is still very limited (1–4), therefore new strategies are warranted. Combining ICI with peptide vaccination strategies could be efficacious since these approaches address synergistic pathways and the combination of both therapies could therefore unleash blockade of effector T-cells and thereby potentially improve anti-tumor outcome.

Dendritic cells (DC) are an attractive target for the initiation of strong immune responses as they possess the capability of capturing antigens and presenting them to specific T-cells for their activation (5, 6). The skin harbors different types of antigen presenting cells, such as dermal DC (dDC) and Langerhans cells (LC) and is therefore an attractive site for delivery of vaccines (7–10). Delivery of vaccines to DC and LC can be addressed by targeting e.g., C-type lectin receptors such as Dendritic Cell-Specific Intercellular adhesion molecule-3-Grabbing Non-integrin (DC-SIGN) and Langerin. This can be easily done using their natural glycan ligands, including Lewis Y (Le^Y^) (11–14). DC have an additional central role when it comes to activation of invariant natural killer T-cells (iNKT) by presentation of glycolipids such as alpha galactosylceramide (αGC) in CD1d (15). iNKT are of special interest in vaccination strategies since they release a wide variety of pro-inflammatory cytokines upon activation, which aids activation of CD8^+^ cytotoxic T-cells, NK-cells B-cells and DC (16–18). For this reason they are widely known as typical strong bridgers of innate and adaptive immune responses (19, 20) and numerous pre-clinical studies have emphasized the potential of iNKT activation in anti-tumor responses (21–24). Interestingly, not only moDC but also dDC play an important role in the stimulation of iNKT. We have previously shown that injected αGC is taken up by skin antigen presenting cells (APC) and results in activation of iNKT which enhances antigen cross-presentation by dDC to provide tumor protection (25). Although the potential of iNKT activation in anti-tumor therapies has been widely acknowledged in experimental models, clinical trials with iNKT have thus far revealed that the induction of effective and durable anti-tumor responses is still very limited (26–31). Therefore, more potent strategies are warranted.

In this study we aimed to develop a Le^Y^-conjugated liposome-based DC targeting vaccine to initiate strong immune responses by induction of tumor antigen specific CD8^+^ specific T-cells and iNKT activation. We show simultaneous melanoma antigen-specific CD8^+^ effector T-cell and iNKT activation by skin emigrated DC upon intradermal delivery of the vaccine, demonstrating its utility for effective cancer vaccination.

## Results

### Liposomes Are Efficiently Internalized by moDC

**Figure 1:**
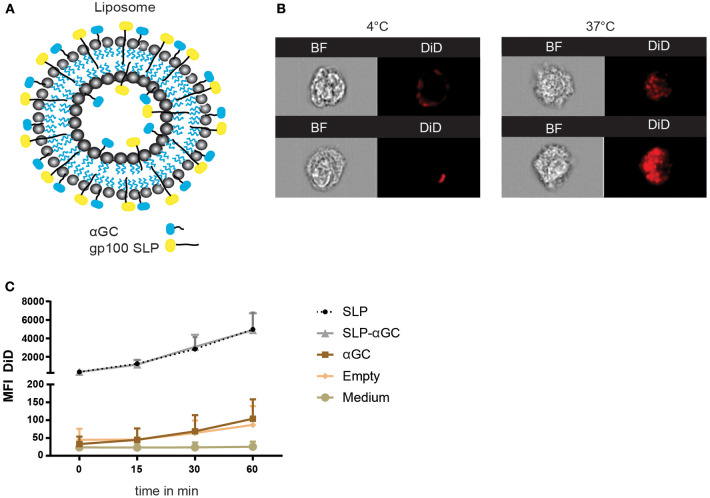
Liposome characteristics determine uptake capacity of moDC. **(A)** Schematic overview of liposomes. Liposomes contain a core of phospholipids and cholesterol, and were loaded with palmitoylated SLP (indicated in yellow), αGC (indicated in blue) or combinations of both components which resided in the lipid bilayer. **(B)** Representative pictures of bright-field (BF) and DiD signal in moDC over time after incubation with 100 μM SLP/αGC liposomes for 45 min at 4°C (left panel) and 60 min at 37°C (right panel). **(C)** Detection of MFI from DiD labeled liposomes over time in human moDC. t=0 represents incubation of moDC for 45 min at 4°C, while *t* = 15, *t* = 30 and *t* = 60 represent MFI after incubation at 37°C. Data is presented as mean ± SEM *n* = 3.

Liposomes were generated using a mixture of phospholipids and cholesterol to form so called empty liposomes. In addition, liposomes were generated with palmitoylated synthetic long peptides (SLP) of MHC class I and II epitopes of the melanoma antigen gp100, αGC and a combination of SLP and αGC that were embedded into the bilayer (Figure 1A). Size distribution with dynamic light scattering analysis and zeta potential were measured and showed equal size distribution and charge independent of liposomal content between formulations (Table 1). To assess the capacity of moDC to internalize our nano-carriers, they were incubated with the different liposomes that were fluorescently labeled with the lipophilic dye DiD and flow cytometry was performed. Analysis with imaging flow cytometry showed clearly that after 45 min incubation at 4°C (*t* = 0), most liposomes were located at the membrane of the DC, but after transfer to 37°C, allowing metabolic activity of the DC and rearrangement of the actin filaments, liposomes were mainly localized intracellularly (Figure 1B). Analysis of DiD signal in moDC as a measurement for uptake of liposomes revealed that moDC most efficiently took up liposomes containing only SLP or a combination of SLP and αGC (Figure 1C) and that the fluorescent content increased over time, suggesting that the amount of liposomes applied did not saturate DC within the given time. In conclusion, liposomes are quickly internalized by moDC.

**Table 1:** Mean size, polydispersity index, and Z potential with SD of five different batches of liposomes.

**Liposome content**	**Size in nm (SD)**	**Polydispersity (SD)**	**Z Potential mV (SD)**
Empty	201.1 (10.8)	0.03 (0.02)	−56.7(3.9)
SLP	190.3 (10.4)	0.11 (0.04)	−62.8(3.9)
αGC	209.4 (16.5)	0.07 (0.01)	−56.8(3.7)
SLP αGC	196.0 (15.0)	0.17 (0.08)	−65.8(3.1)
SLP αGC Le^Y^	180.2 (17.4)	0.17 (0.04)	−53.9(3.0)

### Liposome Loaded moDC Present Antigen to CD8^+^ T-cells and αGC to iNKT

**Figure 2:**
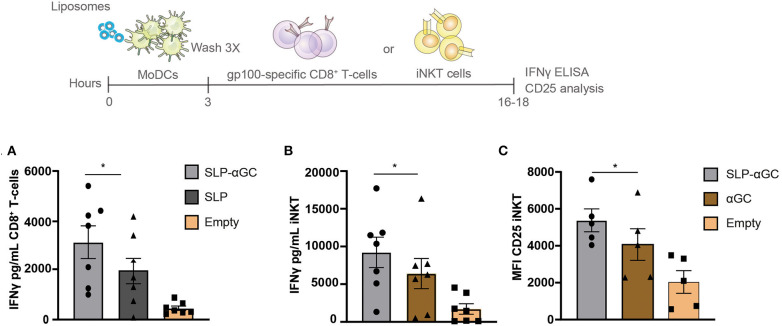
Liposome-loaded moDC present antigen to CD8^+^ T-cells and αGC to iNKT**. (A)** gp100 specific CD8^+^ T-cell activation measured as IFNγ secretion after co-culture with moDC that were loaded with 100 μM liposomes. Data represents mean ± SEM of *n* = 7. **(B)** Representation of IFNγ secretion of iNKT and **(C)** CD25 expression after co-culture with 100 μM liposome-loaded moDC. Data is presented as mean ± SEM *n* = 7 for 2B and *n* = 5 for 2C. Statistical significance based on repeated measures one-way ANOVA with Tukey's *post hoc* test, * < 0.05.

After determining the uptake capacity of liposomes by moDC we aimed to investigate the immune related functional properties of the nano-carrier. Hereto, gp100 SLP/αGC containing liposomes were loaded onto moDC and co-cultured with respectively, gp100 specific CD8^+^ T-cells or iNKT to test for their potential to activate these T-cell subsets. moDC were shown to present the gp100 epitope from the liposomes, activating gp100 specific CD8^+^ T-cells as reflected by the high amounts of secreted IFNγ (Figure 2A). Interestingly, addition of αGC increased IFNγ secretion by CD8^+^ T-cells, even in the absence of iNKT. This might reflect DC activation induced by adjuvant properties of αGC which has been described before (16). When we analyzed the iNKT potentiating capacity of the gp100 SLP/αGC liposomes we observed that after co-culture with iNKT, moDC properly presented encapsulated αGC to iNKT leading to strong IFNγ secretion (Figure 2B) and induction of CD25 expression (Figure 2C). Remarkably, liposomes containing SLP and αGC gave the highest induction of IFNγ secretion and CD25 expression, which is probably not a direct effect of the SLP itself, but through enhanced uptake of liposomes in the presence of SLP as seen in uptake experiments (Figure 1C). Overall, liposomes that included SLP and αGC have a strong potential to activate both CD8^+^ T- and iNKT cells upon DC uptake, indicating that our nano-carrier provides dual activation properties of both the innate and adaptive arm of the immune system.

### Incorporation of Le^Y^ as Targeting Ligand for DC-SIGN and Langerin Increases Uptake of Liposomes in moDC and dDC

**Figure 3:**
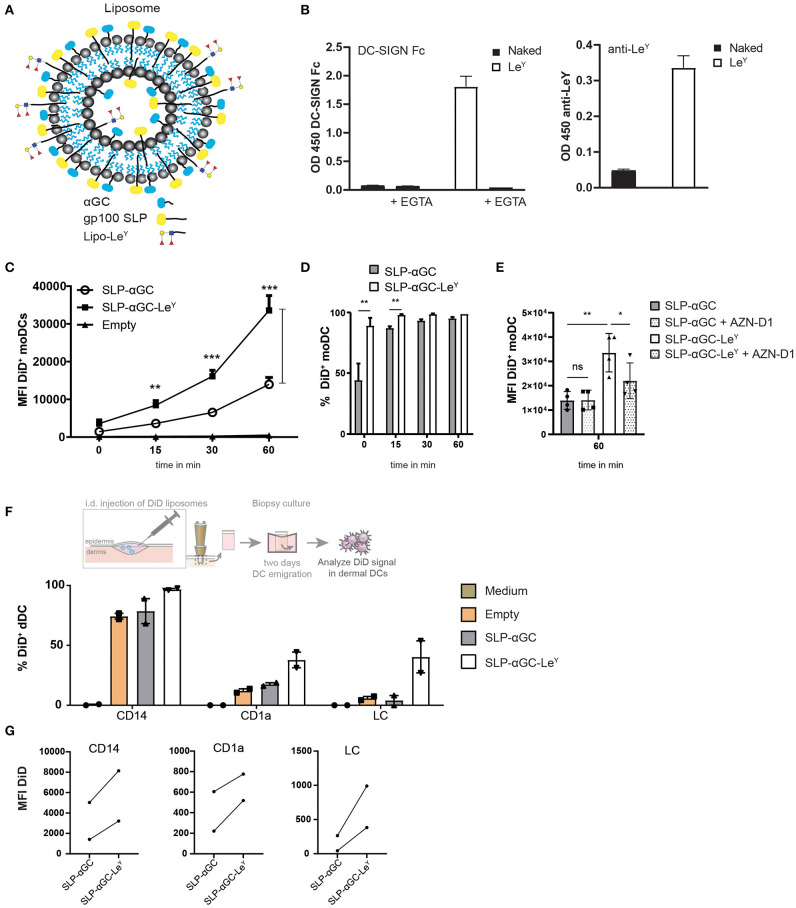
Modification of liposomes with palmitoylated Le^Y^ enhances uptake in moDC and human skin DC. **(A)** Schematic overview of liposomes with Le^Y^. Palmitoylated Le^Y^ is integrated in the lipid bilayer together with SLP and αGC. **(B)** Representative data of *n* = 3, showing binding of DC-SIGN Fc chimera and anti-Le^Y^ antibody to liposomes using binding ELISA. **(C)** Flow cytometry data on moDC loaded with DiD^+^ liposomes over time. *t* = 0 represents MFI after 45 min incubation at 4°C, while *t* = 15, *t* = 30 and *t* = 60 are measured after transfer to 37°C. Data shown as mean ± SEM *n* = 4, one-way ANOVA Tukey's *post hoc* test, ** < 0.01, *** < 0.001. **(D)** Percentages of DiD^+^ moDC after 45 min incubation with liposomes at 4°C (*t* = 0) or after transfer to 37°C (*t* = 15, *t* = 30, *t* = 60). Data represents mean ± SD *n* = 3, paired *t*-test, ** < 0.01. **(E)** Blocking effect of DC-SIGN blocking antibody AZN-D1 on liposome uptake in moDC after 60 min incubation at 37°C. Data represents mean ± SD *n* = 4. Statistical significance based on one-way ANOVA, Sidak's *post hoc* test, * < 0.05, ** < 0.01. **(F)** Representation of % DiD^+^ cells as a measure for uptake by different DC subsets in human skin of two donors. **(G)** MFI of DiD^+^ cells in different human skin subsets of two donors.

To determine whether the immunogenicity of our nano-carrier could be further improved by enabling cutaneous DC targeting and subsequent cross-presentation, we set out to target the C-type lectin receptors DC-SIGN and Langerin on dDC and LC, respectively, by their carbohydrate ligand Lewis Y (Le^Y^). To this end, liposomes were generated and incorporated into the liposome with palmitoylated Le^Y^ (Figure 3A). Attaching Le^Y^ to palmitic hydrazide to form lipo-Le^Y^, allows for integration into the bilayer of the liposome for multivalent presentation and is thereby easily accessible for Le^Y^ binding receptors. First, the presence of Le^Y^ was analyzed by performing binding ELISA using DC-SIGN-Fc and anti-Lewis antibody which confirmed that liposomes had incorporated Le^Y^ (Figure 3B). Addition of EGTA to chelate calcium (Ca2^+^) and thereby prevent Ca2^+^ dependent binding of DC-SIGN-Fc, resulted in complete loss of binding. As hypothesized, incorporation of liposomes with Le^Y^ strongly enhanced uptake of liposomes in DC-SIGN^+^ moDC as indicated by the increase in mean fluorescent intensity (MFI) (Figure 3C). Interestingly, the difference in percentage of DiD^+^ cells was most distinct at *t* = 0 at 4°C, where 90% of the moDC were DiD positive after loading with Le^Y^ modified liposomes compared to 45% after loading with unmodified liposomes (Figure 3D). However, after transfer of moDC to 37°C, it appeared that almost 100% of the moDC also internalized the non-targeted liposomes. Nevertheless, considering the big difference in MFI after 60 min (Figure 3C), we concluded that in presence of the DC targeting ligand Le^Y^, the amount of liposomes per DC was highly increased. Next it was investigated whether this increase in particle uptake could be attributed to DC-SIGN targeting. Hereto, moDC were incubated with a DC-SIGN blocking antibody AZN-D1 and decreased uptake of Le^Y^ decorated liposomes was observed, indicating that uptake was DC-SIGN-mediated (Figure 3E). Next, liposomes were injected intradermal (i.d.) in human skin explants and after 48 hrs. emigrated DC were analyzed for DiD signal (Figure 3F) and gated for DC subset specificity (Figure S1). Also here, Le^Y^ liposomes were taken up more efficiently than unmodified liposomes (Figure 3F). CD14^+^ dDC from human skin took up most liposomes as reflected by the highest percentage and highest MFI (Figure 3G). However, also in CD1a^+^ and LC we could detect a higher frequency of DiD positive cells and increased MFI after injection of Le^Y^ modified liposomes compared to unmodified liposomes. This suggests that Langerin, which also has specificity for Le^Y^, might be the targeted receptor in LC and therefore responsible for the observed increase in uptake. Overall we can conclude that addition of Le^Y^ into the bilayer of liposomes enhances uptake in moDC and multiple subsets of skin APCs.

### Incorporation of Le^Y^ in Liposomes Enhances CD8^+^ T-cell and iNKT Activation

**Figure 4:**
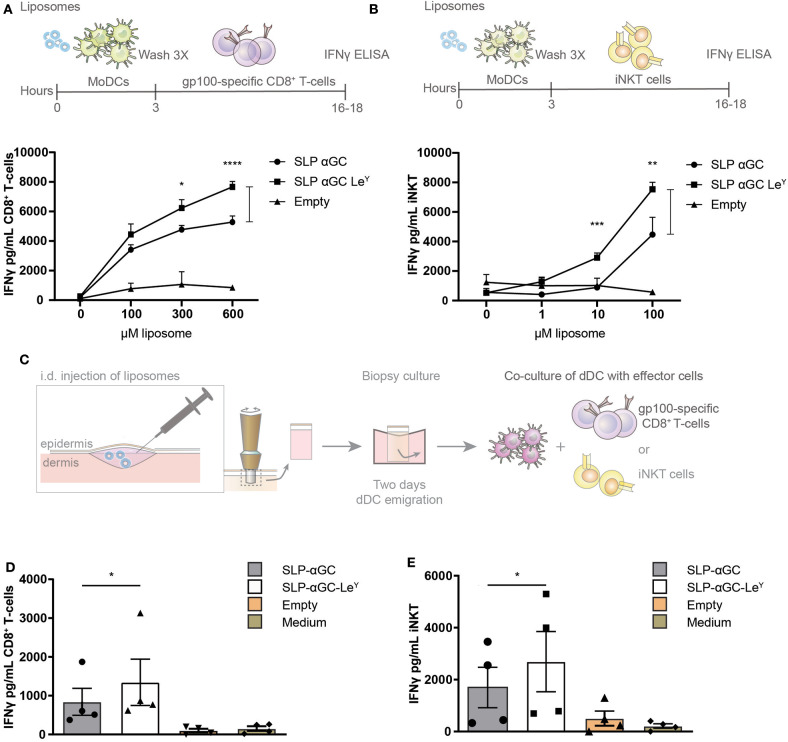
Incorporation of Le^Y^ in liposomes increases CD8^+^ T-cell and iNKT activation by liposome-exposed moDC and human skin DC. **(A)** Data showing activation of gp100 specific T-cells as measure of IFNγ secretion after co-culture with moDC loaded with different concentrations of non-targeting and targeting Le^Y^ liposomes. Concentrations used as indicated in the figure. Data shown as mean ± SD of technical triplicate and representative of *n* = 5, one-way ANOVA, Tukey's *post hoc* test, * < 0.05 **** < 0.0001. **(B)** Secretion of IFNγ by human iNKT cells after co-culture of moDC with unmodified and Le^Y^ liposomes. Concentrations used as indicated in the figure. Data shown as mean ± SD of technical triplicate and representative of *n* = 5, one-way ANOVA, Tukey's *post hoc* test, ** < 0.01 *** < 0.001. **(C)** Schematic overview of experiment set-up for gp100 specific T-cell and iNKT activation by emigrated DC after liposome injection in human skin explants. Migrated skin DC were co-cultured with gp100 specific CD8^+^ T-cell and iNKT for 16–18 h after which IFNγ was measured in supernatant **(D)** IFNγ secretion of gp100 specific CD8^+^ T-cells after co-culture with a mixture of migrated skin-emigrated DC. Data represents mean ± SEM *n* = 4, ratio paired *t*-test, * < 0.05). **(E)** IFNγ release by iNKT after co-culture with a mixture of migrated skin-emigrated DC. Data is shown as mean ± SEM *n* = 4, ratio paired *t*-test, * < 0.05.

Next, it was investigated if enhanced uptake as a result of Le^Y^ incorporation would result in stronger activation of both iNKT and CD8^+^ cellular responses. Therefore, moDC were loaded with gp100 SLP/αGC liposomes containing Le^Y^ as targeting moiety and co-cultured with gp100 specific CD8^+^ T-cells or iNKT. Indeed, both CD8 T-cell (Figure 4A) and iNKT (Figure 4B) activation was increased when moDC were loaded with Le^Y^ liposomes as reflected by increased secretion of IFNγ. The cross-presentation of antigen in Le^Y^ modified liposomes by skin DC was investigated next. Liposomes were injected i.d. and 2-day migrated dermal DC were collected and co-cultured with either gp100 specific CD8^+^ T-cells or iNKT (Figure 4C). Remarkably, also here incorporation of Le^Y^ into the bilayer of the liposomes enhanced presentation of gp100 peptide by skin DC to activate gp100 specific CD8^+^ T-cells (Figure 4D). Additionally, IFNγ production by iNKT after co-culture of dermal DC migrated from the explant injected with Le^Y^ liposomes significantly outperformed the conditions where unmodified liposomes were injected (Figure 4E). In conclusion, incorporation of Le^Y^ enhanced uptake of liposomes by moDC and dDC which concomitantly increased activation of iNKT and CD8^+^ T-cells and thereby displayed potential as a tool for boosting anti-tumor responses.

### Antigen From Liposomes With Incorporated Le^Y^ Are Presented by DC to Activate Primary T-cells

**Figure 5:**
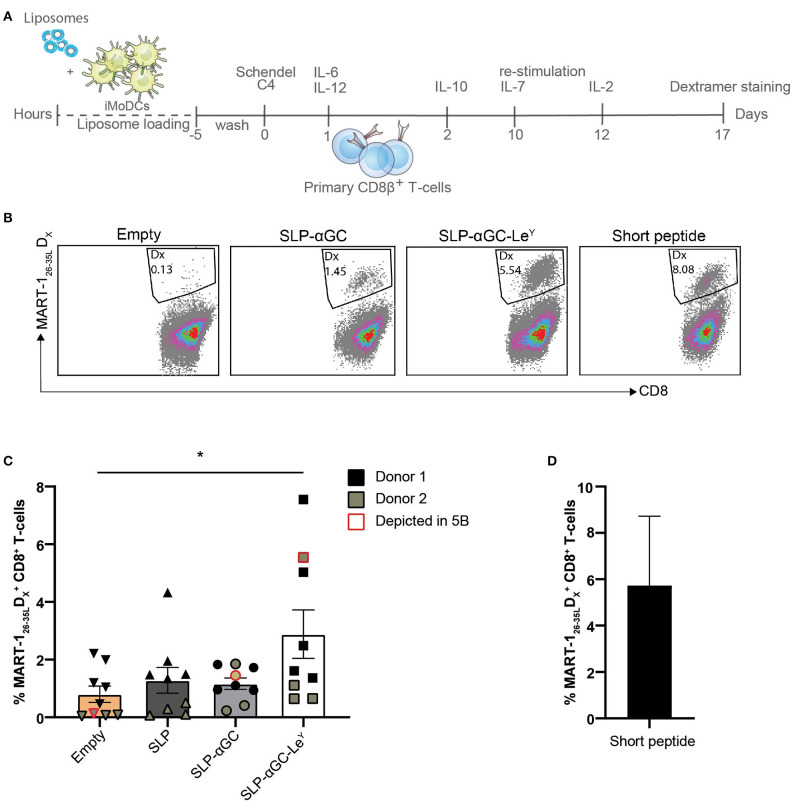
Le^Y^ liposome-loaded DC activate primary T-cells. **(A)** Experimental set-up for priming of T-cells through co-culture of mature DC loaded with MART-1 containing and DC-targeted or non-targeted liposomes. **(B)** Representative dot plots showing MART-1_26−35L_ dextramer positive CD8^+^ T-cells induced by Empty, SLP-αGC, SLP-αGC-Le^Y^ liposomes and short MART-1_26−35L_ peptide 17 days after induction. **(C)** Percentages of MART-1_26−35L_ dextramer positive T-cells after co-culture with liposome-loaded and matured DC 17 days after induction. Five wells of liposome-loaded DC and primary CD8β T-cells were initiated for donor 1 (black) and four bulks for donor 2 (olive green). Data points outlined with red indicate the cultures shown as dot plot in figure 5B. Data represents mean ± SEM, one-way ANOVA Tukey's *post hoc* test, * < 0.05. **(D)** Frequency of MART-1 specific T-cells present in control conditions after co-culture with DCs loaded with short MART-1_26−35L_ peptide.

Next, we assessed whether DC loaded with SLP and αGC containing liposomes could cross-prime CD8^+^ T-cells and if proliferation of T-cells would be enhanced after inclusion of Le^Y^ into the lipid bilayer of liposomes. Since many healthy individuals harbor a high frequency of circulating naïve MART-1 specific T-cells that are characterized by a typical CD28^+^CD45RA^hi^/RO^−^ naïve phenotype (32), we have used this as a model system to study the cross-priming capacity of DC after loading with liposomes. Hereto, a 15-mer MART-1 (Melan A) SLP was incorporated in the liposomes as model antigen. CD8β T-cells were isolated from PBMC and co-cultured with liposome loaded and matured moDC for 10 days after which T-cells were re-stimulated for another 7 days with liposome loaded and matured DC and outgrowth of MART-1_26−35L_ specific CD8^+^ T-cells was measured by HLA-A2 dextramer (D_x_) staining (Figures 5A,B). Highest rates of primed MART-1_26−35L_ reactive T cells were detected in the condition where moDC were loaded with liposomes containing the Le^Y^ glycan. In fact this was the only condition that was significantly different compared to the control condition where moDC were loaded with empty liposomes (Figure 5C). In cultures without Le^Y^, minimal outgrowth was observed. As positive control, DC were loaded with MART-1_26−35L_ short synthetic peptide (Figure 5D). To conclude, moDC loaded with SLP-αGC-Le^Y^ containing liposomes were able to demonstrably cross-prime primary MART-1 specific T-cells, while moDC loaded with all the other liposomes did not. These data thus indicate that targeting with Le^Y^ could potentially facilitate improved CTL cross-priming.

## Discussion

Immune checkpoint inhibition unleashes already existing specific T-cell responses against tumor epitopes in T cell-infiltrated tumors by lifting molecular brakes imposed by the tumor-conditioned tissue microenvironment (33–35). However, more recent pre-clinical studies have shown the importance of the generation and recruitment of *de novo* responses that together with ICI could provide a greater anti-tumor efficacy, especially in so-called “immunological desert” tumors that lack lymphocyte infiltrates (36, 37). Novel vaccination strategies represent a promising approach to convert these immunologically “silent” tumors into effector T cell-infiltrated “hot” tumors. In this study we have generated a liposome-based vaccine with incorporated palmitoylated SLP containing MHC-I and MHC-II epitopes of the melanoma associated antigen gp100, the glycolipid αGC and the palmitoylated natural ligand for C-type lectin receptors DC-SIGN and Langerin, i.e., lipo-Le^Y^. We showed that these 200 nm sized, negatively charged liposomes efficiently delivered SLP and αGC to both moDC, dDC, and LC. We have shown that delivery to moDC and skin DC can be enhanced by inclusion of the targeting moiety Le^Y^ into the liposomal bilayer. As a result of enhanced uptake we could detect improved T-cell responses with potential anti-tumor activity, not only by activation of a gp100 specific CD8^+^ T-cell clone, but also by activation of iNKT and priming of MART-1_26−35L_ reactive T-cells. Therefore, Le^Y^ modified liposomes provide a promising vaccination platform for the induction of multifactorial anti-tumor immunity.

To the best of our knowledge this is the first report using palmitoylated tumor antigens and palmitoylated Le^Y^ in combination with αGC as components in liposomes. We have shown that palmitoylated antigens incorporated in liposomes are efficiently cross-presented to CD8^+^ T-cells and that inclusion of palmitoylated Le^Y^ increases activation of CD8^+^ T-cells as a result of enhanced antigen loading in DC. Evidence is emerging that vaccination with neoepitopes might elicit stronger anti-tumor responses (38–41) than vaccination with common shared tumor antigens and combination of multiple antigens in one vaccine formulation has been reported to efficiently elicit a multi-targeted T cell response (41, 42). Addition of a single palmitic acid is a relatively inexpensive and non-laborious process that can be applied to both antigens and DC-targeting ligands. Therefore, our strategy of incorporating palmitoylated antigens opens a window for development of a personalized vaccine in which identified tumor specific neo-epitopes could be included in the form of SLP, palmitoylated and potentially combined in liposomes as vaccine delivery vehicle to DC by targeting any receptor that might be deemed appropriate.

Interestingly, enhanced uptake of Le^Y^ modified liposomes was not only observed in dDC subsets but also in LC. It is well-know that DC-SIGN and Langerin, which are present on dermal DC and LC respectively, share glycan binding specificity for Le^Y^ (43) but the fact that uptake in LC is observed with palmitoylated Le^Y^ modified liposomes emphasizes that multivalency of the ligand and spatial orientation influences internalization capacity: as reported previously, Langerin mediated uptake of glycan modified liposomes with a thiol-maleimide linker in LC could not be observed (44), whereas glycan-dendrimers bearing Le^Y^, could perfectly target both DC-SIGN and Langerin (45). Apparently, spatial orientation of Le^Y^ in the lipid bilayer of liposomes influences Langerin binding and Langerin-mediated internalization of the vehicle. These results therefore indicate that not only size of the particle, but, more importantly, also the presentation and/or conjugation of glycans on vaccination vehicles such as liposomes or dendrimers should be considered for dual targeting of DC-SIGN and Langerin and the design of glyco-vaccines in general.

For both gp100 specific CD8^+^ T-cells and iNKT, responses were increased after co-culture with SLP-αGC-Le^Y^ loaded DC. However, it remains to be determined whether this increase in IFNγ secretion is due to the fact that frequency of activated cells is higher or that the magnitude of the activation is increased, i.e., secretion of IFNγ per cell. We have earlier reported that stimulation of DC with a different vaccine modality, modified with Le^Y^, induced a higher percentage of degranulating gp100 specific CD8^+^ T-cells, based on CD107a/b cell surface expression (45). Therefore, we speculate that the activation in number of T-cells is higher after co-culture with SLP-αGC-Le^Y^ loaded DC rather than an elevated production of cytokine per cell.

As a result of enhanced uptake, increased gp100 specific CD8^+^ T-cell activation was observed after i.d. injection of Le^Y^ modified liposomes in human skin. This indicates that targeting DC-SIGN and/or Langerin results in effective cross presentation of antigen by skin DC to T-cells. Interestingly, all DC subsets (CD14^+^, CD1a^+^, LC) in skin were taking up Le^Y^ liposomes to a larger extent than unmodified liposomes. Since DC-SIGN is mostly expressed on CD14^+^ cells (13, 45) and they have been described to be either monocyte derived macrophages (46) or macrophage-like cells trans-differentiated from cutaneous CD1a^+^ DC (47–49), it is no surprise that the highest MFI and percentage of DiD^+^ cells could be observed in this subset as a result of their phagocytic capacity and their relatively high expression of DC-SIGN. Although CD14^+^ cells in skin have been defined to be inferior antigen presenters compared to LC and CD1a^+^ cells, they are not incapable of inducing proliferation of allogeneic CD4^+^ and CD8^+^ T-cells (50) and they also have been shown to induce expansion of MART-1^+^ specific T-cells (9) and cross present antigen to CD8^+^ specific gp100 T-cells (13). Additionally their role in activation of memory T-cells has been acknowledged (46). Also in CD1a^+^ dDC, which have a lower expression of DC-SIGN compared to CD14^+^ cells (13, 45), the percentage of DiD^+^ cells increased upon injection of Le^Y^ modified liposomes. Interestingly, Menares et al. proposed a model in which the counterpart of human CD1a dDC in mice, CD11b^+^ DC, mature and migrate to draining lymph nodes (LN) upon tissue resident memory T-cell (T_rm_) activation (51). This might especially be of interest if besides a tumor specific SLP and αGC, another skin memory epitope is included in the vaccine as additional natural adjuvant, whereby CD14^+^ cells potentially could locally activate T_rm_ to in their turn activate CD1a^+^ dDC to carry tumor antigens to the LN for subsequent activation of cytotoxic T-cells. Overall, we think that developing a vaccine that targets multiple skin APC subsets, which all have different functions and advantageous features in the induction of strong anti-tumor immunity, might be a strategic and effective approach for therapeutic vaccination against cancer.

Although little uptake of αGC liposomes could be observed at 60 min, iNKT activation assays with moDC and dDC showed clear iNKT responses. This suggests that little αGC is needed for proper iNKT activation, which is supported by multiple papers reporting that IFNγ secretion could already be measured with doses as low as 100 ng/mL (52, 53). The enhanced effect in activation can be attributed to increased loading of αGC in CD1d as a result of increased uptake in moDC and dDC. The question remains which subset in the skin is responsible for the enhanced iNKT activation after injection of Le^Y^ liposome in human skin. Since CD1d expression on LC is inhibited by TGFβ secretion of keratinocytes (54) and because no expression could be observed in epidermal cell suspensions (55), it is unlikely that the iNKT activation observed after co-culture with DC from liposome injected human skin can be attributed to LC. More likely, iNKT activation after injection in skin can be attributed to one of the dermal HLA-DR^+^ APC subsets (55).

The fact that enhanced activation of iNKT was observed upon co-culture with moDC and dDC, which were loaded with αGC and SLP containing and Le^Y^-modified liposomes, is promising for future *in vivo* studies that yet need to be conducted and will show whether the combination of CD8^+^ T-cells and iNKT activation will lead to enhanced anti-tumor efficacy. Of note, it has already been shown that co-delivery of tumor antigen and αGC can promote strong anti-tumor responses in a prophylactic and therapeutic setting (56). The combination of αGC and SLP in one vaccine formulation evoked strong antigen specific CTL and could either delay or even prevent tumor progression (57, 58). Additionally, multiple studies showed for the efficacy of PD1/PDL-1 blocking antibodies in terms of overcoming attenuation of iNKT activation (59, 60). Therefore, combining liposomes containing SLP and αGC with ICI might also elicit a stronger and more durable iNKT response. Whether iNKT activation induced via delivery of αGC encapsulated in SLP and Le^Y^ containing liposomes to DC also leads to increased anti-tumor responses *in vivo* still needs to be determined.

Different clinical trials with peptide vaccines have shown the efficacy of adjuvants like Montanide and Freud's adjuvant (61–63). Also addition of Addavax and poly I:C or other TLR ligands like CpG to peptide vaccines has shown good efficacy in pre-clinical models (64–67). Furthermore, the role of αGC as adjuvanting component has been extensively described in multiple studies (68–72). Likewise, we demonstrated that inclusion of αGC in liposomes enhanced CD8^+^ T-cell responses compared to liposomes containing SLP alone and that responses of CD8^+^ T-cells but also of iNKT can be further enhanced by addition of Le^Y^. The question remains how these immune responses compare to SLP vaccination with other existing adjuvants. Remarkably Dölen et al. investigated the effect of OVA-αGC nanoparticles (NPs) in comparison to OVA NPs with conventional TLR ligands R848 and poly I:C and found that OVA-αGC outperformed TLR containing NPs (58). Having a direct comparison of αGC NP to other existing adjuvants would be of great value to better understand and implement αGC in SLP containing vaccines. Moreover, it would be very interesting to resolve whether immune responses induced by SLP-αGC-Le^Y^ liposomes could be further enhanced by inclusion of additional adjuvanting components. Interestingly, we have shown in mouse models that 2 h after s.c. injection of Addavax mouse DC-SIGN^+^ cells could be found in the skin (73). Therefore, combining SLP-αGC-Le^Y^ liposomes with Addavax might be a great strategy to recruit DC-SIGN^+^ DCs to even further strengthen antigen specific immune responses.

In summary, combining palmitoylated antigen and palmitoylated Le^Y^ together with a αGC in one liposome constitutes a promising peptide vaccination platform for *in situ* delivery to skin APCs which allows for increased uptake and antigen presentation to CD8^+^ T-cells together with iNKT activation and therefore may contribute to improve ICI anti-cancer immunotherapy.

## Materials and Methods

### Antibodies

CD1a (Clone HI149, BD Biosciences, New Jersey, U.S.), CD14 (Clone M5E2 Sony, San Jose, U.S.), CD141 (Clone 1A4 RUO, BD Biosciences), HLA-DR (Clone G46-6, BD Biosciences), EPCAM (Clone EBA-1, BD Biosciences), anti-Vα24 (Clone C15, Beckman Coulter, Brea, U.S.) anti-TCR Vβ11 (Clone C21 Beckman Coulter), CD8 (clone RPA-T8, BD Biosciences), CD8β (Clone 2ST8.5H7, Beckman Coulter) Fixable Viability Dye (FVD) eFluor 780 (Invitrogen, Waltham, U.S.).

### Peptide Synthesis

The antigenic peptides gp100 (VTHTYLEPGPVTANRQLYPEWTEAQRLDC), MART-1 (YTTAEELAGIGILTV) containing the immune dominant epitope MART-1_26−35L_ and MART-1_26−35L_ (ELAGIGILTV) were synthesized by solid phase peptide synthesis using Fmoc chemistry on a Symphony peptide synthesizer (Protein Technologies Inc., Tucson, U.S.). In the last step of the synthesis, prior to the cleavage of the peptide from the resin, a palmitic tail was added at the N-terminus of the gp100 and MART-1 peptide through a reaction with palmitic anhydride in dichloromethane (DCM). The resulting lipopeptides were cleaved and purified on a preparative Ultimate 3000 HPLC system (Thermo Fisher) over a VYDAC 214MS1022 C4 25 × 250 mm column (Grace Davidson). Mass and purity were confirmed by UPLC-MS on a Ultimate 3000 UHPLC system (Thermo Fisher, Waltham, U.S.) hyphenated with a LCQ-Deca XP Iontrap ESI mass spectrometer (Thermo Finnigan, Waltham, U.S.) using a Prosphere HP C4 5 μm 150 × 4.6 mm column and ionizing the sample in positive mode.

### Preparation of Lewis^Y^ Glycolipid

Lewis Y (Le^Y^)-hexadecanohydrazide (palmitic hydrazide) ea. palmitoylated Le^Y^ (from here on, shortly, lipo-Le^Y^) was prepared from Le^Y^ pentasaccharide (Elicityl, Crolles, France) and hexadecanohydrazide through a reductive amination reaction. Addition of CHCl_3_/MeOH/H_2_O at 8:1:8 v/v ratio, followed by vigorous stirring and centrifugation, allowed the extraction of lipo-Le^Y^ as a white slurry at the interphase. The slurry was freeze-dried to remove residual solvent. The correct mass of the glycolipid was confirmed by ESI-MS (Thermo Finnigan LCQ-Deca XP Iontrap mass spectrometer in positive mode) using nanospray capillary needle.

### Liposome Preparation

Liposomes containing palmitoyl-gp100 or palmitoyl-MART-1 lipopeptides and lipo-Le^Y^ targeting glycan were prepared from a mixture of phospholipids and cholesterol utilizing the film extrusion method. Briefly, EPC-35 (Lipoid):EPG-Na (Lipoid):Cholesterol (Sigma-Aldrich, St. Louis, U.S.) at a ratio of 3.8:1:2.5 in mol were dissolved in a mixture of chloroform/methanol. The lipophilic fluorescent tracer DiD (1′-dioctadecyl-3,3,3′,3′-tetramethyl indodicarbocyanine; Life Technologies, 0.1% in mol) was added to the mixture (where indicated) as well as palmitoyl-gp100 (400 μg) or palmitoyl-MART-1 (400 μg), lipo-Le^Y^ (1.5 mg) and the NKT cell activator α-galactosylceramide, KRN7000 (Funakoshi, Tokyo, Japan) (30 μg). A lipid film was obtained by evaporation of the solvent under reduced pressure at 50°C that was hydrated in HEPES buffer solution pH 7.5 yielding a liposome preparation of phospholipids at a concentration of ~8 μmol total lipid/mL. After extrusion through two stacked polycarbonate filters of 200 nm, the non-encapsulated antigen and lipo-Le^Y^ were washed away by two consecutive ultracentrifugation steps on Beckman Ultracentrifuge at 55.000 rpm. The final suspension of liposomes was obtained in Hepes buffer pH 7.5. After 15 min of vigorous stirring and overnight incubation at 4°C, the liposome suspensions were centrifuged at 55.000 rpm and resuspended in Hepes buffer pH 7.5 twice. Size, polydispersity index, zeta potential and amount of encapsulated fluorochrome DiD was determined prior to use, as previously described (74). After destruction with perchloric acid, phospholipid content (concentration in molar) was quantified spectrophotometrically as described before (75). The amount of liposomes used in the experiments was calculated based on the phospholipid contents in mol as previously described (76). Comparison of lipid concentration before (8.7 mM) and after (5.6 mM) extrusion revealed an overall yield of 64%. The content of αGC and lipopeptide was calculated from the phospholipid concentration. The concentration of αGC and lipo-SLP was 19.2 μg/mL and 256 μg/mL, respectively.

### Generation of moDC

Peripheral blood mononuclear cells (PBMCs) were isolated from buffy coats of healthy volunteers (Sanquin, Amsterdam, The Netherlands) by centrifugation on a Ficoll gradient. First, blood was gently mixed with PBS containing 1% citrate and carefully layered on top of Ficoll. After 30 min centrifugation at 700 × g, the interphase containing monocytes and lymphocytes was collected. Next, monocytes and lymphocytes were washed with PBS/Citrate at 400 × g for 10 min and the pellet was resuspended in complete RPMI. To isolate monocytes, PBMCs were carefully added on top of a Percoll layer (GE Healthcare, Chicago, U.S.) at a concentration of 10 × 10^6^ cells/mL and centrifuged at 400 × g for 40 min. Again the interphase was collected and tubes were filled with PBS/Citrate prior to a 10 min centrifugation at 400 × g. After washing three times with PBS/Citrate, pellets were resuspended in complete RPMI. MoDC were generated by culturing monocytes for 5–7 days at a concentration of 1.25^*^10^6^/mL in complete RPMI (Lonza, Basel, Switzerland) containing 500 μ/mL IL-4 (ImmunoTools, Friesoythe, Germany) and 800 μ/mL Granulocyte Macrophage Colony stimulating Factor (GM-CSF) (ImmunoTools) using T75 flasks (Greiner, Kremsmünster, Austria).

### Isolation and Culture of iNKT

iNKT cell lines were generated from PBMCs of healthy donors by magnetic activated cell sorting (MACS). iNKT were enriched by using a murine anti-human TCR Vα24 mAb in combination with magnetic beads labeled with a polyclonal goat-anti –mouse Ab (Miltenyi, Bergisch Gladbach, Germany). Initial iNKT expansion was achieved by co-culturing iNKT for 7 days with αGC 100 ng/mL (Funakoshi) loaded and LPS (Sigma Aldrich, 100 ng/mL) pulsed moDC in the presence of cytokines rhIL-7 10 μ/mL and rhIL-15 10 ng/mL. Purification of iNKT was achieved by repeating magnetic sorting with the murine anti-human TCR Vβ11 mAb. For evaluation of iNKT purity, cells were incubated for 30 min at 4°C with FITC-labeled anti-human TCR Vα24 mAb and PE-labeled anti-human TCR Vβ11 mAb, washed twice and analyzed by flow cytometry on a FACS BD LSRFortessa based on double positive TCR Vα24/ Vβ11 selection. Purified (Vα24^+^ Vβ11^+^ cells) iNKT were weekly re-stimulated with irradiated feeder-mix, consisting of allogeneic PBMCs from two healthy donors and JY cells, an EBV transformed lymphoblastoid cell line, in Yssel's medium supplemented with 50U/mL rhIL-2 (Proleukin, Clinigen, Burton upon Trent, U.K.) and 50 ng/mL PHA (Vector Laboratories). iNKT purity used for all experiments was >90%.

### Intradermal Injection of Liposomes and Culture of Skin Biopsies

Liposomes were diluted in serum-free IMDM (Lonza) supplemented with antibiotics (50 U/mL penicillin, 50 μg/mL streptomycin and 10 μg/mL gentamycin) and injected intradermal in human abdominal skin explants from healthy donors (Bergman Clinics, Bilthoven, The Netherlands) in a volume of 20 μl as previously described (13). Human skin samples were obtained after informed consent from patients that underwent corrective breast or abdominal plastic surgery, according to hospital guidelines and in accordance with the “Code for Proper Use of Human Tissues” as formulated by the Dutch Federation of Medical Scientific Organizations. Biopsies of 8 mm were taken with a biopsy punch (Microtec, Zutphen, The Netherlands) and cultured in a 48-well plate in 1 mL/well complete IMDM (10% FCS, 2 mM L-glutamine, 100 U/mL penicillin (Lonza), 100 U/mL streptomycin (Lonza) and 10 μg/mL gentamycin). After 48 h of culture, biopsies were removed from wells and crawl-out DC were collected, pooled and used for experiments.

### Liposome Binding to and Uptake via DC-SIGN in moDC

Liposomes labeled with the lipophilic dye DiD (Invitrogen), were added at a concentration of 100 μM to moDC, which were either pre-incubated with 20 μg/mL of a neutralizing αDC-SIGN antibody (AZN-D1 in house production) for 30 min at 37°C or left untreated before loading. Loading of moDC with labeled liposomes continued for 45 min at 4°C after which a moDC sample was collected and washed two times with PBS prior to fixation on ice with 4% PFA (Aurion) for 20 min (*t* = 0). Immediately after collecting the *t* = 0 sample, the remaining moDC were incubated at 37°C for 15 (*t* = 15), 30 (*t* = 30), or 60 (*t* = 60) minutes. Samples were washed three times with PBS containing 1% BSA (PBA) and fixed in 4% PFA before analysis on LSR Fortessa (BD Biosciences).

### Liposome Uptake by dDC

DiD labeled liposomes containing palmitoylated gp100 peptide, αGC and Le^Y^ were diluted in serum-free IMDM (Lonza), supplemented with antibiotics (50 U/mL penicillin, 50 μg/mL streptomycin and 10 μg/mL gentamycin), and injected intradermal in human abdominal skin explants from healthy donors (Bergman Clinics, Bilthoven, The Netherlands) in a volume of 20 μl. Next, 8 mm biopsies were taken with a biopsy punch (Microtec) and biopsies were cultured in a 48-well plate (Greiner) in 1 mL complete IMDM (10% FCS, 2 mM L-glutamine, 50 U/mL penicillin, 50 μg/mL streptomycin and 10 μg/mL gentamycin). After 48 hrs. of culture, biopsies were removed from wells and crawled out dDC were collected and stained with a multi-color antibody panel containing anti-CD1a, anti-CD14, anti-HLA-DR, anti-EPCAM, FVD.

### Antigen Presentation of moDC to CD8^+^ gp100 Specific T-cells

Day 5–7 moDC or skin migrated DC (20.000/well) were incubated with different concentrations of liposomes containing gp100 SLP, αGC and Le^Y^ in triplicate for 3 h at 37°C in a 96-well U bottom plate in a total volume of 100μl. After incubation with the liposomes, DC were washed three times with serum-free RPMI and centrifuged for 3 min at 400 × g. gp100-specific CD8^+^ T-cells [generated and cultured as described previously (77)] were added to the liposome-loaded moDC or migrated dDC/LC at a concentration of 100.000 cells/well (ratio 1:5) in enriched IMDM, i.e., Yssel's medium (20 μg/mL human transferrin, 5 μg/mL insulin, 2 μg/mL linoleic acid, 2 μg/mL palmitic acid, 0.25% BSA, 1.8 μg/mL 20-amino ethanol). The DC and T-cell co-culture was left overnight at 37°C/5%CO_2_ and subsequently 75 μl supernatant was collected for IFNγ ELISA. Samples were stored at −20°C.

### iNKT Activation Assay

Day 5–7 moDC or skin migrated DC (20.000/well) were incubated with different concentrations of liposomes containing gp100 SLP, αGC and Le^Y^ in triplicate for 3 h at 37°C in a 96-well U bottom plate in a total volume of 100 μl. After incubation with the liposomes, moDC were washed three times with serum-free RPMI and centrifuged for 3 min at 300 × g. iNKT (isolated and cultured as described above) were added to the liposome-loaded moDC or migrated skin DC at a concentration of 100.000 cells/well (ratio 1:5) in enriched IMDM (Lonza), i.e., Yssel's medium. The DC and T-cell co-culture was left overnight and subsequently 75 μl supernatant was collected for IFNγ ELISA. Samples were stored at −20°C. CD25 levels were measured by flow cytometry and cells were gated on live Vβ11^+^ Vα24^+^ cells.

### Cytokine ELISA

1 μg/mL anti-human IFNγ (Invitrogen) was coated overnight in 50 mM Na_2_CO_3_, pH 9.7. Plates were washed with PBS containing 0.05% Tween and blocked for 30 min at 37°C with 1% BSA in PBS. Supernatants were added together with the corresponding IFNγ detection antibody for 2 h at RT while shaking. Presence of the cytokines was detected with Streptavidin-PO (Biosource Finnigan, Waltham, U.S.). Binding was determined by adding substrate buffer (110 mM citric acid (Merck), 110 mM sodium acetate (Fisher scientific, Waltham, U.S.), pH 4 and 100 μg/mL fresh TMB (Sigma) and 0.006% H_2_O_2_. Absorbance was measured at 450 nm on an ELISA reader (Bio-Rad Benchmark, Hercules, U.S.).

### Priming of MART Specific CTLs

PBMCs isolated from a buffy coat (Sanquin) were pre-stained with an unlabeled CD8β^+^ mAb and incubated with magnetic polyclonal goat-anti–mouse beads (Miltenyi). CD8β^+^ CTLs, were positively selected by MACS sorting. Monocytes were isolated from the CD8β- fraction through the use of CD14 magnetic sorting beads (Miltenyi) and in complete RPMI (Lonza) containing 500 U/mL IL-4 (ImmunoTools) and 800 U/mL GM-CSF (ImmunoTools) for 5 days. At day 5, immature moDC were pre-cultured in the presence of 25 μM of liposomes and loaded for 5–6 h. Maturation of moDC, was induced by using the “Schendel-C4” maturation cocktail, consisting of the following cytokines: 500IU GM-CSF 5 ng/mL IL4 (Strathmann, Hamburg, Germany), 480IU/mL TNFα (Miltenyi), 10 ng/mL IL-1β (Miltenyi), 250 ng/mL PGE2 (Sigma), 1000IU/mL IFNγ (Calbiochem, Darmstadt, Germany) and 1 μg/mL R848 (Invivogen). As a control mature moDC were loaded with 1 μg/mL MART-1_26−35L_ short 9mer synthetic peptide (EAAGIGILTV) in the presence of β2-microglubolin (Fitzgerald Industries, Acton, US) for 4 h. Ten day cultures were started with 1 × 10^5^ liposome- or peptide-loaded mature moDC, 1 × 10^6^ CD8β^+^ CTLs and 1 × 10^6^ CD8β- autologous irradiated (50Gy) feeder cells, in Yssel's medium supplemented with IL-12 10 ng/mL (Novartis, Basel, Switzerland) and IL-6 10 ng/mL (R&D systems, Minnesota, U.S.) in a 48 wells plate. Twenty-four hours after start of culture IL-10 10 ng/mL (Ebioscience, Waltham, U.S.) was added. At day 10 cultures were re-stimulated with 1x10^5^ liposome- or peptide loaded mature moDC and supplemented with 10 UI/mL IL-7. Fourty-eight hours after re-stimulation 10IU/mL IL-2 (Proleukin) was added to the cultures. The % of MART-1 specific CD8^+^ T cells, was analyzed by flow cytometry using a MART-1_26−35L_ PE-labeled HLA-A2 Dextramer (Immudex, Copenhagen, Denmark), APC-labeled CD8 mAb and FVD.

### Statistics

Statistical analysis was accomplished by the use of Graphpad 8.0.2 Prism software. Data was analyzed using one-way or two-way ANOVA with *post hoc* analysis as indicated in the figure legend or with use of (ratio paired) *t*-test.

## Data Availability Statement

The datasets generated for this study are available on request to the corresponding author.

## Author Contributions

DS, AH, and JV performed research. DS, TG, HV, and YK designed the experiments. Data collection was performed by DS, AH, JV, SD, JL, and RV. SB optimized assays. Products were synthesized by AH, MA, and HK. DS and JV analyzed data. Manuscript was prepared by DS and corrected by YK, TG, and HV. All authors have read the article and gave their approval for publication.

## Conflict of Interest

HV is employed by and acts as chief scientific officer of Lava Therapeutics. The remaining authors declare that the research was conducted in the absence of any commercial or financial relationships that could be construed as a potential conflict of interest.

## References

[1] Hodi FS O'Day SJ McDermott DF Weber RW Sosman JA Haanen JB. Improved survival with ipilimumab in patients with metastatic melanoma. N Engl J Med. (2010) 363:711–23. 10.1056/NEJMoa1003466 20525992 PMC3549297

[2] Larkin J Chiarion-Sileni V Gonzalez R Grob JJ Cowey CL Lao CD Combined nivolumab and ipilimumab or monotherapy in untreated melanoma. N Engl J Med. (2015) 373:23–34. 10.1056/NEJMoa1504030 26027431 PMC5698905

[3] Wolchok JD Chiarion-Sileni V Gonzalez R Rutkowski PJ Grob J Cowey CL. Overall survival with combined nivolumab and ipilimumab in advanced melanoma. N Engl J Med. (2017) 377:1345–56. 28889792 10.1056/NEJMoa1709684 PMC5706778

[4] Robert C Thomas L Bondarenko I O'Day S Weber J Garbe C. Ipilimumab plus dacarbazine for previously untreated metastatic melanoma. N Engl J Med. (2011) 364:2517–26. 10.1056/NEJMoa1104621 21639810

[5] Banchereau J Steinman RM. Dendritic cells and the control of immunity. Nature. (1998) 392:245–52. 10.1038/32588 9521319

[6] Steinman RM Banchereau J. Taking dendritic cells into medicine. Nature. (2007) 449:419–26. 10.1038/nature06175 17898760

[7] Nestle FO Zheng XG Thompson CB Turka LA Nickoloff BJ. Characterization of dermal dendritic cells obtained from normal human skin reveals phenotypic and functionally distinctive subsets. J Immunol. (1993) 151:6535. 7504023

[8] Lenz A Heine M Schuler G Romani Human N. and murine dermis contain dendritic cells. Isolation by means of a novel method and phenotypical and functional characterization. J Clin Investig. (1993) 92:2587–96. 10.1172/JCI116873 8254016 PMC288454

[9] Klechevsky E Morita R Liu M Cao Y Coquery S Thompson-Snipes L. Functional specializations of human epidermal langerhans cells and cd14+ dermal dendritic cells. Immunity. (2008) 29:497–510. 10.1016/j.immuni.2008.07.013 18789730 PMC2688399

[10] Gunawan M Jardine L Haniffa M. Isolation of human skin dendritic cell subsets. Methods Mol Biol. (2016) 1423:119–28. 10.1007/978-1-4939-3606-9_8 27142012

[11] van Dinther D Stolk DA van de Ven R van Kooyk Y de Gruijl TD. Targeting C-type lectin receptors: a high-carbohydrate diet for dendritic cells to improve cancer vaccines. J Leukoc Biol. (2017) 102:1017–34. 10.1189/jlb.5MR0217-059RR 28729358 PMC5597514

[12] Garcia-Vallejo JJ Unger WW Kalay H van Kooyk Y. Glycan-based DC-SIGN targeting to enhance antigen cross-presentation in anticancer vaccines. Oncoimmunology. (2013) 2:e23040. 10.4161/onci.23040 23525136 PMC3601176

[13] Fehres CM van Beelen AJ Bruijns CM Ambrosini M Kalay H Bloois LV. *In situ* delivery of antigen to DC-SIGN+CD14+ dermal dendritic cells results in enhanced CD8+ T-cell responses. J Investig Dermatol. (2015) 135:2228–36. 10.1038/jid.2015.152 25885805

[14] Horrevorts SK Stolk DA Ven RV Hulst MB Hof VH Duinkerken S. Glycan-modified melanoma-derived apoptotic extracellular vesicles as antigen source for anti-tumor vaccination. Cancers. (2019) 11:1266. 10.3390/cancers11091266 31466401 PMC6769957

[15] Spada FM Borriello F Sugita MG Watts FM Koezuka Y Porcelli SA. Low expression level but potent antigen presenting function of CD1d on monocyte lineage cells. Eur J Immunol. (2000) 30:3468–77. 10.1002/1521-414130:12<3468::AID-IMMU3468>3.0.CO;2-C 11093166

[16] Fujii S.-I. Shimizu K Smith C Bonifaz L Steinman RM. Activation of natural killer T cells by alpha-galactosylceramide rapidly induces the full maturation of dendritic cells in vivo and thereby acts as an adjuvant for combined CD4 and CD8 T cell immunity to a coadministered protein. J Exp Med. (2003) 198:267–79. 10.1084/jem.20030324 12874260 PMC2194082

[17] Hermans IF Silk JD Gileadi U Salio M Mathew B Ritter G. NKT cells enhance CD4+ and CD8+ T cell responses to soluble antigen *in vivo* through direct interaction with dendritic cells. J Immunol. (2003) 171:5140–7. 10.4049/jimmunol.171.10.5140 14607913

[18] Carnaud C Lee D Donnars O Park SH Beavis A Koezuka Y. Cutting edge: Cross-talk between cells of the innate immune system: NKT cells rapidly activate NK cells. J Immunol. (1999) 163:4647–50. 10528160

[19] Fujii S Liu K Smith C Bonito AJ Steinman RM. The linkage of innate to adaptive immunity via maturing dendritic cells *in vivo* requires CD40 ligation in addition to antigen presentation and CD80/86 costimulation. J Exp Med. (2004) 199:1607–18. 10.1084/jem.20040317 15197224 PMC2212806

[20] Van Kaer L Parekh VV Wu L. Invariant natural killer T cells: bridging innate and adaptive immunity. Cell Tissue Res. (2011) 343:43–55. 10.1007/s00441-010-1023-3 20734065 PMC3616393

[21] Swann JB Uldrich AP van Dommelen S Sharkey J Murray WK Godfrey DI. Type I natural killer T cells suppress tumors caused by p53 loss in mice. Blood. (2009) 113:6382–5. 10.1182/blood-2009-01-198564 19234138 PMC2710930

[22] Bellone M Ceccon M Grioni M Jachetti E Calcinotto A Napolitano A. iNKT cells control mouse spontaneous carcinoma independently of tumor-specific cytotoxic T cells. PLoS One. (2010) 5:e8646. 10.1371/journal.pone.0008646 20072624 PMC2800182

[23] Crowe NY Smyth MJ Godfrey DI. A critical role for natural killer T cells in immunosurveillance of methylcholanthrene-induced sarcomas. J Exp Med. (2002) 196:119–27. 10.1084/jem.20020092 12093876 PMC2194015

[24] Macho-Fernandez E Cruz LJ Ghinnagow R Fontaine J Bialecki E Frisch B. Targeted delivery of alpha-galactosylceramide to CD8alpha+ dendritic cells optimizes type I NKT cell-based antitumor responses. J Immunol. (2014) 193:961–9. 10.4049/jimmunol.1303029 24913977

[25] Bontkes HJ Moreno M Hangalapura B Lindenberg JJ de Groot J Lougheed S. Attenuation of invariant natural killer T-cell anergy induction through intradermal delivery of α-galactosylceramide. Clin Immunol. (2010) 136:364–74. 10.1016/j.clim.2010.04.019 20570567

[26] Giaccone G Punt CJ Ando Y Ruijter R Nishi N Peters M. A phase I study of the natural killer T-cell ligand alpha-galactosylceramide (KRN7000) in patients with solid tumors. Clin Cancer Res. (2002) 8:3702–9. Retrieved from: https://clincancerres.aacrjournals.org/12473579

[27] Motohashi S Nagato K Kunii N Yamamoto H Yamasaki K Okita K. A phase I-II study of alpha-galactosylceramide-pulsed IL-2/GM-CSF-cultured peripheral blood mononuclear cells in patients with advanced and recurrent non-small cell lung cancer. J Immunol. (2009) 182:2492–501. 10.4049/jimmunol.0800126 19201905

[28] Exley MA Friedlander P Alatrakchi N Vriend L Yue S Sasada T. Adoptive transfer of invariant NKT cells as immunotherapy for advanced melanoma: a phase i clinical trial. Clin Cancer Res. (2017) 23:3510–9. 10.1158/1078-0432.CCR-16-0600 28193627 PMC5511564

[29] Yamasaki K Horiguchi S Kurosaki M Kunii N Nagato K Hanaoka H. Induction of NKT cell-specific immune responses in cancer tissues after NKT cell-targeted adoptive immunotherapy. Clin Immunol. (2011) 138:255–65. 10.1016/j.clim.2010.11.014 21185787

[30] Richter J Neparidze N Zhang L Nair S Monesmith T Sundaram R. Clinical regressions and broad immune activation following combination therapy targeting human NKT cells in myeloma. Blood. (2013) 121:423–30. 10.1182/blood-2012-06-435503 23100308 PMC3548165

[31] King LA Lameris R de Gruijl TD van der Vliet HJ CD1d-invariant natural killer T cell-based cancer immunotherapy: alpha-galactosylceramide and beyond. Front Immunol. (2018) 9:1519 10.3389/fimmu.2018.01519 30013569 PMC6036112

[32] Pittet MJ Valmori D Dunbar PR Speiser DE Lienard D Lejeune F. High frequencies of naive Melan-A/MART-1-specific CD8(+) T cells in a large proportion of human histocompatibility leukocyte antigen (HLA)-A2 individuals. J Exp Med. (1999) 190:705–15. 10.1084/jem.190.5.705 10477554 PMC2195613

[33] Tumeh PC Harview CL Yearley JH Shintaku IPE Taylor JM Robert L. PD-1 blockade induces responses by inhibiting adaptive immune resistance. Nature. (2014) 515:568–71. 10.1038/nature13954 25428505 PMC4246418

[34] Ji RR Chasalow SD Wang L Hamid O Schmidt H Cogswell J. An immune-active tumor microenvironment favors clinical response to ipilimumab. Cancer Immunol Immunother. (2012) 61:1019–31. 10.1007/s00262-011-1172-6 22146893 PMC11028506

[35] Li J Byrne KT Yan F Yamazoe T Chen Z Baslan T. Tumor cell-intrinsic factors underlie heterogeneity of immune cell infiltration and response to immunotherapy. Immunity. (2018) 49:178–93.e7. 10.1016/j.immuni.2018.06.006 29958801 PMC6707727

[36] Yost KE Satpathy AT Wells DK Qi Y Wang C Kageyama R. Clonal replacement of tumor-specific T cells following PD-1 blockade. Nat Med. (2019) 25:1251–9. 10.1038/s41591-019-0522-3 31359002 PMC6689255

[37] Carreno BM Magrini V Becker-Hapak M Kaabinejadian S Hundal J Petti AA. Cancer immunotherapy. A dendritic cell vaccine increases the breadth and diversity of melanoma neoantigen-specific T cells. Science. (2015) 348:803–8. 10.1126/science.aaa3828 25837513 PMC4549796

[38] D'Alise AM Leoni G Cotugno G Troise F Langone F Fichera I. Adenoviral vaccine targeting multiple neoantigens as strategy to eradicate large tumors combined with checkpoint blockade. Nat Commun. (2019) 10:2688. 10.1038/s41467-019-10594-2 31217437 PMC6584502

[39] Tran E Robbins PF Rosenberg SA. 'Final common pathway' of human cancer immunotherapy: targeting random somatic mutations. Nat Immunol. (2017). 18:255–62. 10.1038/ni.3682 28198830 PMC6295671

[40] Hacohen N Fritsch EF Carter TA Lander ES Wu CJ. Getting personal with neoantigen-based therapeutic cancer vaccines. Cancer Immunol Res. (2013) 1:11. 10.1158/2326-6066.CIR-13-0022 24777245 PMC4033902

[41] Ott PA Hu Z Keskin DB Shukla SA Sun J Bozym DJ. Corrigendum: an immunogenic personal neoantigen vaccine for patients with melanoma. Nature. (2018) 555:402. 10.1038/nature25145 29542692 PMC6064631

[42] Sahin U Derhovanessian E Miller M Kloke BP Simon P Lower M. Personalized RNA mutanome vaccines mobilize poly-specific therapeutic immunity against cancer. Nature. (2017) 547:222–6. 10.1038/nature23003 28678784

[43] Andreini M Doknic D Sutkeviciute I Reina JJ Duan J Chabrol E. Second generation of fucose-based DC-SIGN ligands: affinity improvement and specificity versus Langerin. Org Biomol Chem. (2011) 9:5778–86. 10.1039/c1ob05573a 21735039

[44] Fehres CM Kalay HS Bruijns CMS Musaafir AM Ambrosini M van Bloois L. Cross-presentation through langerin and DC-SIGN targeting requires different formulations of glycan-modified antigens. J Controlled Release. (2015) 203:67–76. 10.1016/j.jconrel.2015.01.040 25656175

[45] Duinkerken S Horrevorts SK Kalay H Ambrosini M Rutte L de Gruijl TD. Glyco-dendrimers as intradermal anti-tumor vaccine targeting multiple skin dc subsets. Theranostics. (2019) 9:5797–809. 10.7150/thno.35059 31534520 PMC6735376

[46] McGovern N Schlitzer A Gunawan M Jardine L Shin A Poyner E. Human dermal CD14+ cells are a transient population of monocyte-derived macrophages. Immunity. (2014) 41:465–77. 10.1016/j.immuni.2014.08.006 25200712 PMC4175180

[47] de Gruijl TD Sombroek CC Lougheed SM Oosterhoff D Buter J van den Eertwegh AJM. A postmigrational switch among skin-derived dendritic cells to a macrophage-like phenotype is predetermined by the intracutaneous cytokine balance. J Immunol. (2006) 176:7232. 10.4049/jimmunol.176.12.7232 16751366

[48] van de Ven R Lindenberg JJ Oosterhoff D de Gruijl TD. Dendritic cell plasticity in tumor-conditioned skin: CD14(+) cells at the cross-roads of immune activation and suppression. Front Immunol. (2013) 4:403. 10.3389/fimmu.2013.00403 24324467 PMC3839226

[49] Klechevsky E Banchereau J. Human dendritic cells subsets as targets and vectors for therapy. Ann N Y Acad Sci. (2013) 1284:24-30. 10.1111/nyas.12113 23651190

[50] Haniffa M Shin A Bigley V McGovern N Teo P See P. Human tissues contain CD141hi cross-presenting dendritic cells with functional homology to mouse CD103+ nonlymphoid dendritic cells. Immunity. (2012) 37:60–73. 10.1016/j.immuni.2012.04.012 22795876 PMC3476529

[51] Menares E Gálvez-Cancino F Cáceres-Morgado P Ghorani E López E Díaz X. Tissue-resident memory CD8+ T cells amplify anti-tumor immunity by triggering antigen spreading through dendritic cells. Nat Commun. (2019) 10:4401. 10.1038/s41467-019-12319-x 31562311 PMC6765014

[52] Ishikawa A Motohashi S Ishikawa E Fuchida H Higashino K Otsuji M. A Phase I study of α-galactosylceramide (KRN7000)–pulsed dendritic cells in patients with advanced and recurrent non–small cell lung cancer. Clin Cancer Res. (2005) 11:1910. 10.1158/1078-0432.CCR-04-1453 15756017

[53] Hogan AE O'Reilly V Dunne MR Dere RT Zeng SG O'Brien C. Activation of human invariant natural killer T cells with a thioglycoside analogue of α-galactosylceramide. Clin Immunol. (2011) 140:196–207. 10.1016/j.clim.2011.03.016 21493160

[54] Ronger-Savle S Valladeau J Claudy A Schmitt D Peguet-Navarro J Dezutter-Dambuyant C. TGFβ inhibits CD1d expression on dendritic cells. J Investig Dermatol. (2005) 124:116–8. 10.1111/j.0022-202X.2004.23315.x 15654963

[55] Gerlini G Hefti HP Kleinhans M Nickoloff BJ Burg G Nestle FO. CD1d is expressed on dermal dendritic cells and monocyte-derived dendritic cells. J Investig Dermatol. (2001) 117:576–582. 10.1046/j.0022-202x.2001.01458.x 11564162

[56] Ghinnagow R De Meester J Cruz LJ Aspord C Corgnac S Macho-Fernandez E. Co-delivery of the NKT agonist α-galactosylceramide5 and tumor antigens to cross-priming dendritic cells breaks tolerance to self-antigens and promotes antitumor responses. Oncoimmunology. (2017) 6:e1339855. 10.1080/2162402X.2017.1339855 28932640 PMC5599097

[57] Neumann S Young K Compton B Anderson R Painter G Hook S. Synthetic TRP2 long-peptide and α-galactosylceramide formulated into cationic liposomes elicit CD8+ T-cell responses and prevent tumour progression. Vaccine. (2015) 33:5838–44. 10.1016/j.vaccine.2015.08.083 26363382

[58] Dölen Y Kreutz M Gileadi U Tel J Vasaturo A van Dinther EAW. Co-delivery of PLGA encapsulated invariant NKT cell agonist with antigenic protein induce strong T cell-mediated antitumor immune responses. OncoImmunology. (2016) 5:e1068493. 10.1080/2162402X.2015.1068493 26942088 PMC4760331

[59] Kamata T Suzuki A Mise N Ihara F Takami M Makita Y. Blockade of programmed death-1/programmed death ligand pathway enhances the antitumor immunity of human invariant natural killer T cells. Cancer Immunol Immunother. (2016) 65:1477–89. 10.1007/s00262-016-1901-y 27631416 PMC5099366

[60] Parekh VV Lalani S Kim S Halder R Azuma M Yagita H. PD-1/PD-L blockade prevents anergy induction and enhances the anti-tumor activities of glycolipid-activated invariant NKT cells. J Immunol. (2009) 182:2816. 10.4049/jimmunol.0803648 19234176 PMC2709814

[61] Kenter GG Welters MJP Valentijn PM Lowik JG Berends-van der Meer DMA Vloon PG. Vaccination against HPV-16 oncoproteins for vulvar intraepithelial neoplasia. N Engl J Med. (2009) 361:1838–47. 10.1056/NEJMoa0810097 19890126

[62] Saavedra D Crombet T. CIMAvax-EGF: A new therapeutic vaccine for advanced non-small cell lung cancer patients. Front Immunol. (2017) 8:269. 10.3389/fimmu.2017.00269 28348561 PMC5346887

[63] Massarelli E William W Johnson F Kies M Ferrarotto R Guo M. Combining immune checkpoint blockade and tumor-specific vaccine for patients with incurable human papillomavirus 16–related cancer: a phase 2 clinical trial. JAMA Oncol. (2019) 5:67–73. 10.1001/jamaoncol.2018.4051 30267032 PMC6439768

[64] Cho HI Barrios K Lee YR Linowski AK Celis E. BiVax: a peptide/poly-IC subunit vaccine that mimics an acute infection elicits vast and effective anti-tumor CD8 T-cell responses. Cancer Immunol Immunother. (2013) 62:787–99. 10.1007/s00262-012-1382-6 23266830 PMC3625508

[65] Zhu X Nishimura F Sasaki K Fujita M Dusak JE Eguchi J. Toll like receptor-3 ligand poly-ICLC promotes the efficacy of peripheral vaccinations with tumor antigen-derived peptide epitopes in murine CNS tumor models. J Transl Med. (2007) 5:10. 10.1186/1479-5876-5-10 17295916 PMC1802742

[66] Lynn GM Sedlik C Baharom F Zhu Y Ramirez-Valdez RA Coble VL. Peptide–TLR-7/8a conjugate vaccines chemically programmed for nanoparticle self-assembly enhance CD8 T-cell immunity to tumor antigens. Nat Biotechnol. (2020) 38:320–32. 10.1038/s41587-019-0390-x 31932728 PMC7065950

[67] Maynard SK Marshall JD MacGill RS Yu L Cann JA Cheng LI. Vaccination with synthetic long peptide formulated with CpG in an oil-in-water emulsion induces robust E7-specific CD8 T cell responses and TC-1 tumor eradication. BMC Cancer. (2019) 19:540. 10.1186/s12885-019-5725-y 31170937 PMC6555006

[68] Nishimura T Kitamura H Iwakabe K Yahata T Ohta A Sato M. The interface between innate and acquired immunity: glycolipid antigen presentation by CD1d-expressing dendritic cells to NKT cells induces the differentiation of antigen-specific cytotoxic T lymphocytes. Int Immunol. (2000) 12:987–94. 10.1093/intimm/12.7.987 10882410

[69] Kitamura H Iwakabe K Yahata T Nishimura S Ohta A Ohmi Y. The natural killer T (NKT) cell ligand alpha-galactosylceramide demonstrates its immunopotentiating effect by inducing interleukin (IL)-12 production by dendritic cells and IL-12 receptor expression on NKT cells. J Exp Med. (1999) 189:1121–8. 10.1084/jem.189.7.1121 10190903 PMC2193012

[70] Teng MW Westwood JA Darcy PK Sharkey J Tsuji M Franck RW. Combined natural killer T-cell based immunotherapy eradicates established tumors in mice. Cancer Res. (2007) 67:7495–504. 10.1158/0008-5472.CAN-07-0941 17671220

[71] Gonzalez-Aseguinolaza G Van Kaer L Bergmann CC Wilson JM Schmieg J Kronenberg M. Natural killer T cell ligand alpha-galactosylceramide enhances protective immunity induced by malaria vaccines. J Exp Med. (2002) 195:617–24. 10.1084/jem.20011889 11877484 PMC2193764

[72] Carreño LJ Kharkwal SS Porcelli SA. Optimizing NKT cell ligands as vaccine adjuvants. Immunotherapy. (2014) 6:309–20. 10.2217/imt.13.175 24762075 PMC4128316

[73] Schetters STT Kruijssen JW Crommentuijn HW Kalay H Ochando J den Haan MM. Mouse DC-SIGN/CD209a as target for antigen delivery and adaptive immunity. Front Immunol. (2018) 9:990. 10.3389/fimmu.2018.00990 29867967 PMC5949514

[74] Joshi MD Unger WW van Beelen AJ Bruijns SC Litjens M van Bloois L. DC-SIGN mediated antigen-targeting using glycan-modified liposomes: formulation considerations. Int J Pharm. (2011) 416:426–32. 10.1016/j.ijpharm.2011.02.055 21371544

[75] Rouser G Fkeischer S Yamamoto A. Two dimensional then layer chromatographic separation of polar lipids and determination of phospholipids by phosphorus analysis of spots. Lipids. (1970) 5:494–6. 10.1007/BF02531316 5483450

[76] Nakamura T Yamazaki D Yamauchi J Harashima H. The nanoparticulation by octaarginine-modified liposome improves α-galactosylceramide-mediated antitumor therapy via systemic administration. J Controlled Release. (2013) 171:216–24. 10.1016/j.jconrel.2013.07.004 23860186

[77] Schaft N Willemsen RA de Vries J Lankiewicz B Essers BW Gratama JW. Peptide fine specificity of anti-glycoprotein 100 CTL is preserved following transfer of engineered TCR alpha beta genes into primary human T lymphocytes. J Immunol. (2003) 170:2186–94. 10.4049/jimmunol.170.4.2186 12574392

