# Supplementary figures and images for: Lipo-Based Vaccines as an Approach to Target Dendritic Cells for Induction of T- and iNKT Cell Responses
Source: Front Immunol. 2020 May 27;11:990. doi: 10.3389/fimmu.2020.00990 (PMC7267035; doi:10.3389/fimmu.2020.00990)

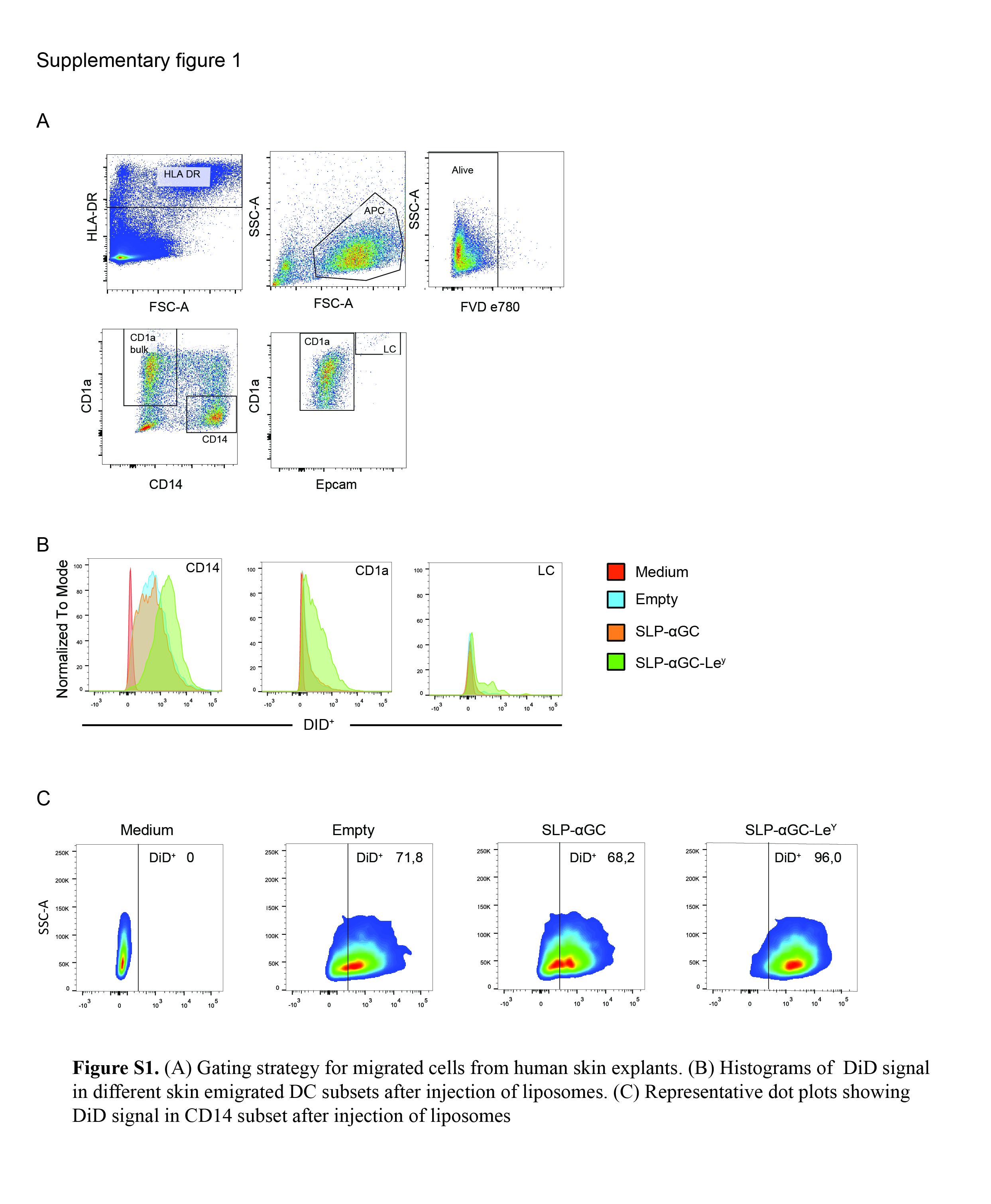

Supplement: Supplementary file 1 [file Image_1.JPEG]
